# Bovine herpesvirus 1 can cross the intact zona pellucida of bovine oocytes after artificial infection

**DOI:** 10.1371/journal.pone.0218963

**Published:** 2019-07-18

**Authors:** Vanessa Lopes Dias Queiroz-Castro, Eduardo Paulino da Costa, Saullo Vinicius Pereira Alves, Mariana Machado-Neves, José Domingos Guimarães, Lidiany Lopes Gomes, Stella Vieira Domingos, Caroline Gomides Ribeiro, Rebeca Toledo Caldas, Abelardo Silva-Júnior

**Affiliations:** 1 Department of Veterinary, Division of Animal Reproduction, Laboratory of Oocyte Maturation and “In Vitro” Fertilization, Federal University of Vicosa, Vicosa, Minas Gerais, Brazil; 2 Department of General Biology, Division of Structural and Cell Biology, Laboratory of Structural Biology, Federal University of Vicosa, Vicosa, Minas Gerais, Brazil; 3 Department of Veterinary, Division of Preventive Medicine and Public Health, Laboratory of Animal Virology, Federal University of Vicosa, Vicosa, Minas Gerais, Brazil; Rowan University, UNITED STATES

## Abstract

Bovine herpesvirus 1 (BHV1) is an important bovine pathogen, responsible for respiratory diseases and reproductive problems. This study investigated the penetration capacity of BHV1 into oocytes after co-incubation for either 1 h or 24 h. Immunofluorescence assays in *cumulus*-oocyte complexes (COCs) and denuded oocytes (without the presence of *cumulus* cells) were performed and evaluated using confocal laser scanning microscopy. Blood samples and ovaries from BHV1 seronegative cows were used. The oocytes recovered were divided into two groups. Group I comprised COCs (n = 312) and denuded oocytes (n = 296), which were experimentally infected with BHV1 and incubated for 1 h at 38.5°C and 5% CO_2_. Group II comprised COCs (n = 425) and denuded oocytes (n = 405), which were co-incubated with BHV1 under the same conditions for 24 h. The negative control of these two groups was respectively subjected to the same protocol, except for exposure to BHV1. To our knowledge, this study provides the first evidence of BHV1 detection within COCs and denuded oocytes exhibiting intact zona pellucida when co-incubated with the virus for 24 h. Immunolocalization also confirmed the presence of BHV1 in the cytoplasm of the *cumulus* cells of all COCs exposed to the virus after both incubation periods. In conclusion, detection of BHV1 inside oocytes has a great meaning for the field of animal reproduction. The detection of BHV1 in different layers of *cumulus* cells also demonstrates that these cells are sources of viral infection.

## Introduction

Bovine herpesvirus 1 (BHV1) is the causative agent of infectious bovine rhinotracheitis (IBR) responsible for important reproductive disorders such as endometritis, oophoritis, temporary infertility, embryonic death, and miscarriages [1,2]. It is thus an economically significant veterinary pathogen involved in financial losses of around US$ 379.00 per infected cow [3]. Overall, *alphaherpesvirinae* viruses, including BHV1, can establish latent infections in neuronal ganglia after primary infection, which may make infected animals lifelong carriers and potential disseminators [4–6]. For instance, the immunosuppression of hosts leads to the re-emergence of BHV1 with episodes of viral re-excretion that contribute to its transmission, as well as impair health management strategies [7,8].

In vitro experiments have shown that cumulus cells are virus potential replication sites [9,10,11]. Our group has recently detected BHV1 in uterus, oviducts, and ovarian tissues from naturally infected and asymptomatic cows, in addition to the cumulus cells [12, 13]. In fact, several layers of this cell type surround the oocyte and form cumulus oocyte complexes (COCs), which play an essential role in maturation, fertilization and early embryo development processes [14,15]. Alves et al. [16] reported that COCs obtained from cows naturally infected with BHV1 harbored virus-positive cumulus cells, and its presence impaired the in vitro nuclear maturation rate.

Several studies have been carried out to determine whether bovine embryos produced in vitro and exposed to BHV1 would be compromised in their initial development. Those findings, however, are still controversial. Guérin et al. [17] observed that oocytes exposed to BHV1 showed reduced rates of in vitro fertilization and cleavage. In addition, Vanroose et al. [11] found that the exposure of oocytes to BHV1 during in vitro maturation resulted in significantly lower rates of blastocyst development. Makarevich et al. [18] noted that embryo exposure to BHV1 compromised embryonic development, with 80% of those embryos stagnating during development and degenerating. Conversely, Bielanski and Dubuc [19] found no effect on the initial development of embryos exposed to the virus. Nevertheless, in all these studies, the authors emphasized that the zona pellucida (ZP) was intact.

Other studies have explored the effectiveness of the ZP as a barrier to viral infection. It is known that the zona pellucida is an extracellular matrix rich in glycoproteins, which separates the oocyte from *cumulus* cells [20]. It may protect embryos against BHV1 infection during the early stages of development when its structure is intact [21–24]. Vanroose et al. [11,22] concluded that BHV1 is not capable of the overpass intact ZP of bovine oocytes and embryos, however, they observed virus replicating within embryo cells when the ZP was previously removed. On that basis, studies emphasized the ZP efficiency as a barrier because no fluorescent microspheres of similar size to BHV1 were able to enter co-incubated zygotes in medium containing the microspheres [23,24]. In contrast, Silva-Frade et al. [25] identified BoHV-5 within bovine oocytes via in situ hybridization assays, concluding that even intact ZP was ineffective in protecting against viral infection.

In this framework, the present study investigated whether BHV1 can trespass on the intact ZP of bovine oocytes in the presence, or absence, of cumulus cells. Oocytes were co-incubated with this virus for one and 24 h and assessed using viral immunolabelling and laser scanning confocal microscopy.

## Material and methods

### Sample collection

Blood samples and ovaries were collected from cows that had not been vaccinated against BHV1 in a slaughterhouse located in the municipality of Muriaé- Minas Gerais, Brazil (21°8'59"S and 42°25'36"W). During the slaughter, blood samples were collected with vacutainer tubes at the time of bleeding. All experimental procedures were conducted in accordance with the ethical principles adopted by the National Council of Animal Experimentation with definitive authorization from the Ethics Committee on Animal Use of the Federal University of Vicosa under protocol n° 94/2015.

### Infected oocytes

COCs were recovered from the ovary by aspiration from follicles 2 to 6mm in diameter. Only COCs with several layers of compact cumulus cells and homogeneous cytoplasm were used. Zona pellucida integrity was assessed using stereoscopic microscopy, as routinely performed during in vitro embryo production system. No morphological changes were observed in the zona pellucida or cumulus cells. The oocytes were transferred to culture plates containing Talp-Hepes medium, and were morphologically classified according to Costa et al. [26]. Other recovered COCs were denuded (*cumulus* cells removed) according to the procedures described by Costa et al. [26]. So far, all oocytes were processed as undefined serological status, since the results of virus neutralization assay were available 72 h later. Seropositive cow oocytes detected by this assay were excluded from the experiment.

Thereafter, oocytes from seronegative cows were divided into two groups. Group I was composed of COCs (n = 312) and denuded oocytes (n = 296) experimentally infected with 10 μL of BHV1 virus 10^4.3^ TCID_50_/mL [25] in 100 μL of maturation medium [27] with virus free tested bovine fetal serum. Its negative control was composed of 273 COCs and 310 denuded oocytes that were subjected to the same protocol, except for the BHV1 exposure. They were then incubated for 1 h at 38.5°C and 5% CO_2_. Group II, in turn, was composed of 425 COCs and 405 denuded oocytes exposed with 10 μL of BHV1 10^4.3^ TCID_50_/mL using the same maturation medium and incubated for 24 h at 38.5°C and 5% CO_2_. Its negative control consisted of 398 COCs and 425 denuded oocytes that were incubated for 24 h in the same conditions with no BHV1 exposure. After the incubation period, COCs and denuded oocytes were evaluated under a stereomicroscope (Olympus, Japan), and later fixed in 4% paraformaldehyde-picric acid solution (Sigma-Aldrich, St. Louis, MO, USA) for assessment by immunofluorescence assays according to Queiroz-Castro et al. [12].

### Cells and viruses

The “Los Angeles” BHV1 strain sample was replicated in Madin-Darby bovine kidney (MDBK) cells cultured in monolayers. The cells were multiplied and maintained at 37°C and 5% CO_2_ atmosphere using minimum essential medium (MEM, Sigma-Aldrich, St. Louis, USA) plus 0.4 mg/L streptomycin (Sigma-Aldrich, St. Louis, USA) and 1.6 mg/L penicillin (Sigma- Aldrich, St. Louis, USA), and supplemented with 10% fetal bovine serum (FBS, Gibco-BRL, Grand Island, USA). The viruses were titrated using the Tissue Culture Infective Dose method (TCID_50_) according to the Reed and Muench method [28].

### Virus neutralization assay

Virus neutralization assays were performed as described by House and Baker [29], with the addition of 100 TCID_50_/50 μL of the LA BHV1 strain to the serum dilutions of each sample. After incubation of the serum-virus for 1 h at 37°C in a CO_2_ incubator, 50 μL of MDBK cell suspension was added at a concentration of 300,000 mL^-1^ cells. Tests were read after 72 h of incubation by monitoring the cytopathic effect. The neutralizing activity of the anti-BHV1 antibody was expressed as the geometric mean of the observed values. Positive and negative reference samples were used as controls.

### Immunolocalization of BHV1

After the incubation period and the stereomicroscope evaluation, all COCs and denuded oocytes of each animal were prepared for immunofluorescence assays, as previously described by Queiroz-Castro et al. [12]. Briefly, samples were transferred to PBS with 1% Triton X-100 (Sigma-Aldrich, St. Louis, MO, USA) for 1 h to promote the permeabilization of the cell membrane. This procedure allows antibody binding to the viral glycoprotein C present in the cell cytoplasm. Thereafter, COCs and denuded oocytes were incubated with a mouse primary monoclonal antibody specific for BHV1 gC glycoprotein (1:100; VMRD, NW, Washington, USA) overnight. Later on, they were incubated with anti-mouse IgG conjugated secondary antibody with fluorescein isothiocyanate-FITC (1:200; Sigma-Aldrich, St. Louis, MO, USA) for 1 h. The nucleic acid dye TO-PRO—3 Iodide (Waltham, Massachusetts, USA) was used for cumulus cells staining. Samples were mounted on slides identified with Mowiol (Sigma-Aldrich, St. Louis, MO, USA), and analyzed on a Zeiss 510 META laser scanning confocal microscope at 200 x or 400 x magnification. An Argon laser (488 nm—excitation) was used to excite FITC fluorophore (490 nm–excitation), whereas the HeNe laser was used to excite TO-PRO—3 Iodide fluorescence (642 nm–excitation). The reconstruction of serial cuts (stepsize of 1 μm) from COCs and denuded oocytes was performed using the z-stack tool from confocal laser scanning microscope, which allows three-dimension image interpretation using LSM Image Browser. Likewise, negative controls in which the primary antibody was omitted were included in immunofluorescence studies.

## Results

**Fig 1:**
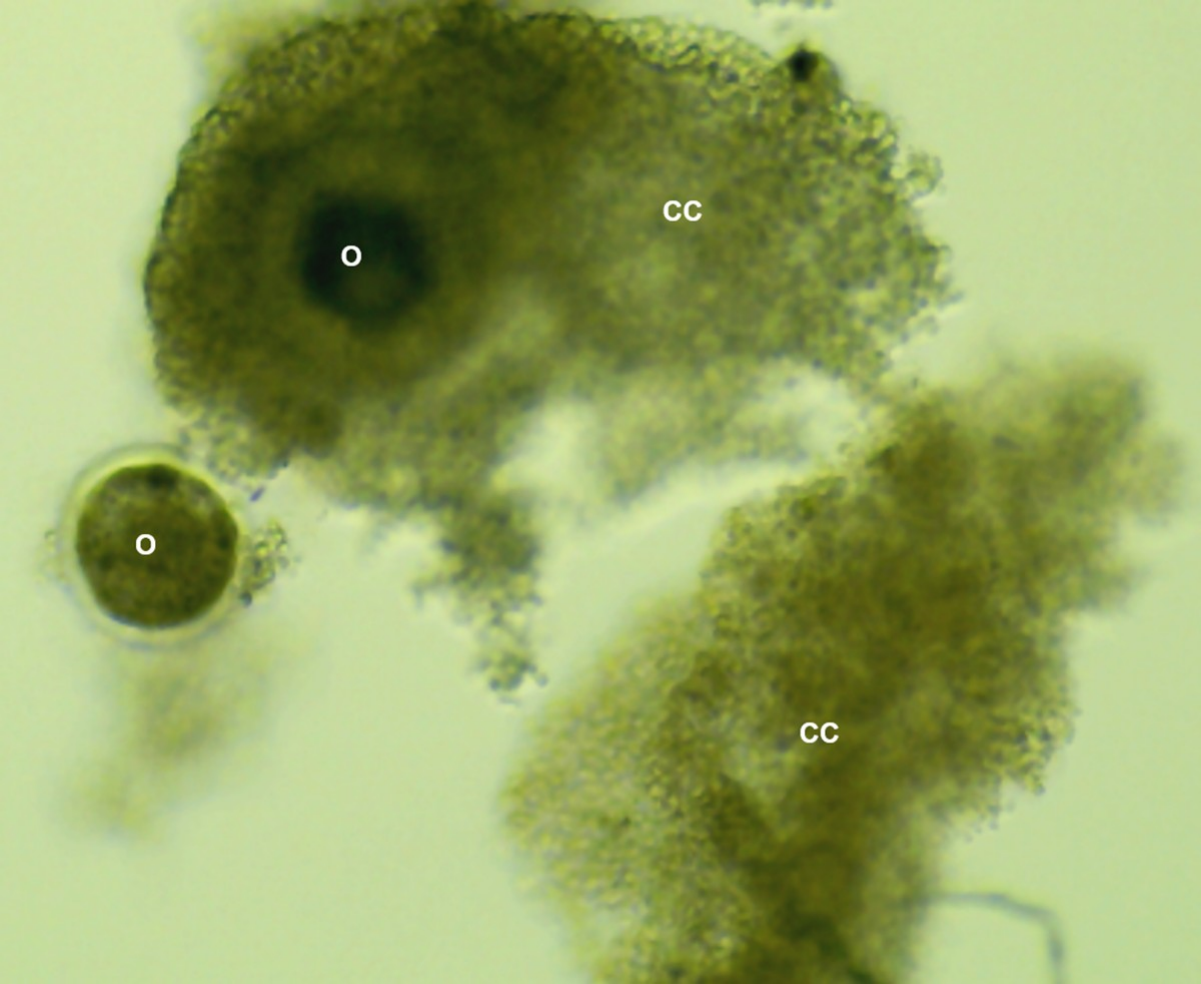
Image of partial disintegration of *cumulus* cells obtained by stereoscope microscopy. Experimental infection of bovine oocytes (O) with BHV1 showing partial disintegration of *cumulus* cells (CC). Magnification of 40x.

Under light microscopy, control COCs (n = 273) and denuded oocytes (n = 310) showed no morphological changes after 1 h incubation with BHV1-free medium. Similarly, control COCs (n = 398) and denuded oocytes (n = 425) presented no changes after 24 h incubation. In contrast, 281 from 312 COCs co-incubated with BHV1 for 1 h showed partial disintegration of *cumulus* cells (Fig 1) (cytopathic effect), whereas all COCs (n = 425) showed partial disintegration of *cumulus* cells after 24 h incubation. The exposure time, however, did not affect the morphological features observed in denuded oocytes exposed to BHV1 after 1 h (n = 296) and 24 h (n = 405).

**Fig 2:**
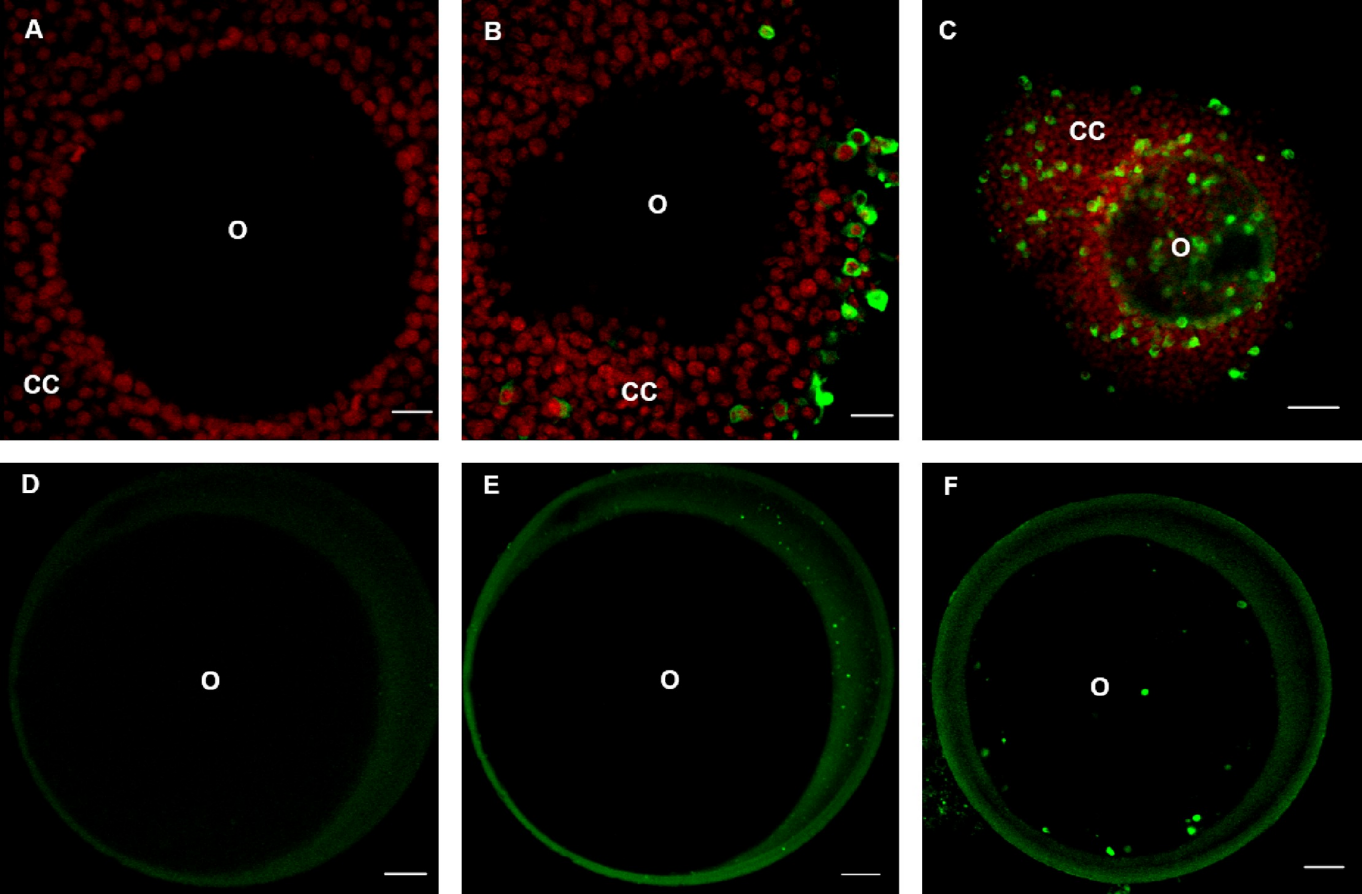
Images of COCs and denuded oocytes obtained by confocal laser scanning microscopy. Experimental infection of bovine oocytes with BHV1 incubated for one and 24 h. (A) confocal image of COC negative control, (B) immunolabelling of BHV1 (green) in the cytoplasm of *cumulus* cells (CC) of co-incubated COCs for 1 h (Scale bar = 20 μm) and (C) immunolabelling of BHV1 in the cytoplasm of COC *cumulus* cells co-incubated for 24 h (Scale bar = 50 μm). O: oocyte. The nuclei of the *cumulus* cells were stained with TO-PRO 3 iodide (red). (D) denuded oocyte control. (E) denuded oocyte co-incubated with BHV1 for 1 h and (F) denuded oocyte co-incubated with BHV1 for 24 h (Scale bar = 20 μm).

**Fig 3:**
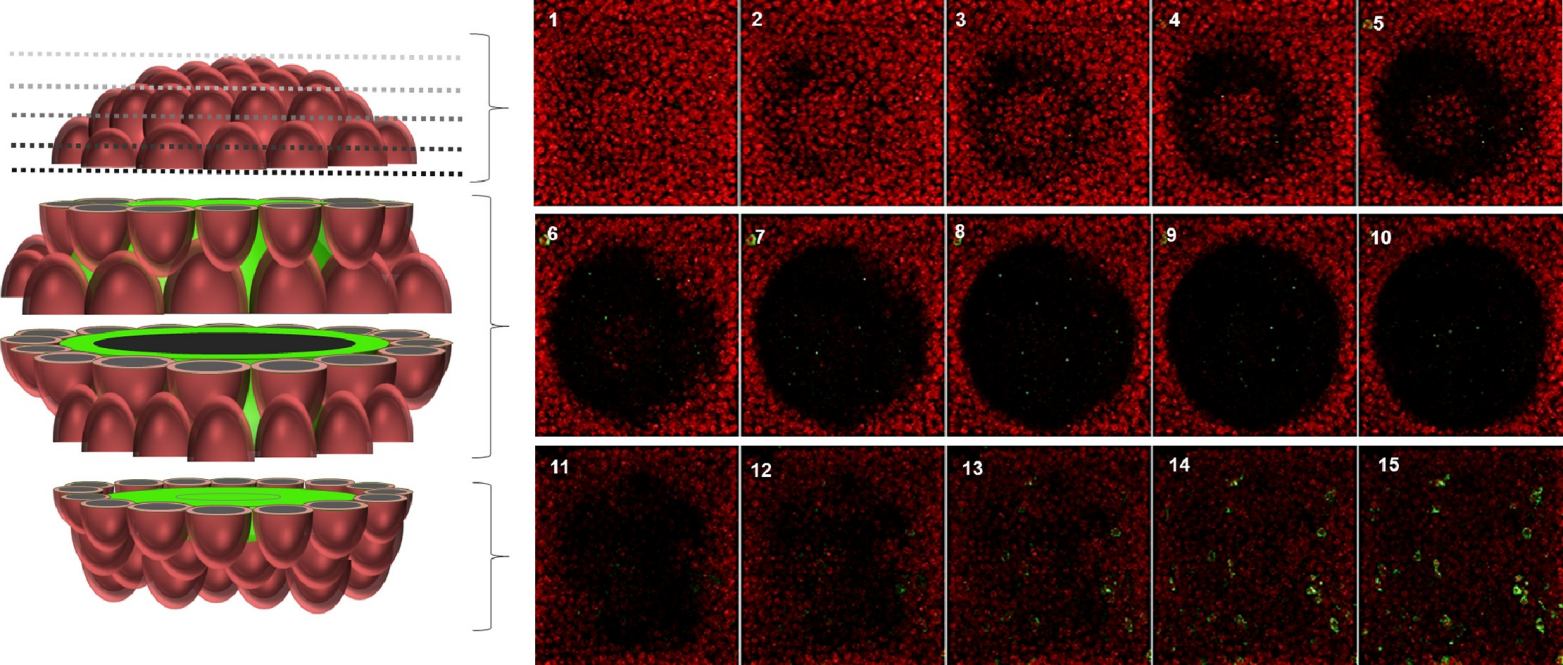
*Cumulus* oocyte complex in a sequence of confocal Z-stack images. Images of COC co-incubated with BHV1 for 24 h in a series of focal planes (step size of 1μm) evidencing the presence of BHV1 (green) inside the oocyte.

As expected, COCs from negative control groups did not present positive-BHV1 gC glycoprotein labeling after 1 h and 24 h incubation under immunofluorescence (Fig 2A). Conversely, all COCs (n = 312) co-incubated for 1 h were BHV1 positive. The virus was detected in the cytoplasm of *cumulus* cells, especially in those located peripherally (Fig 2B). After 24 h incubation with BHV1, additionally, all COCs (n = 425) presented positive labeling for BHV1 gC glycoprotein in the cytoplasm of *cumulus* cells closest to the ZP and inside the oocyte (Fig 2C). The z-stack reconstruction image allows the observation of BHV1 inside the COCs (Fig 3). No secondary antibody non-specific labelling was evidenced in the negative control technique.

**Fig 4:**
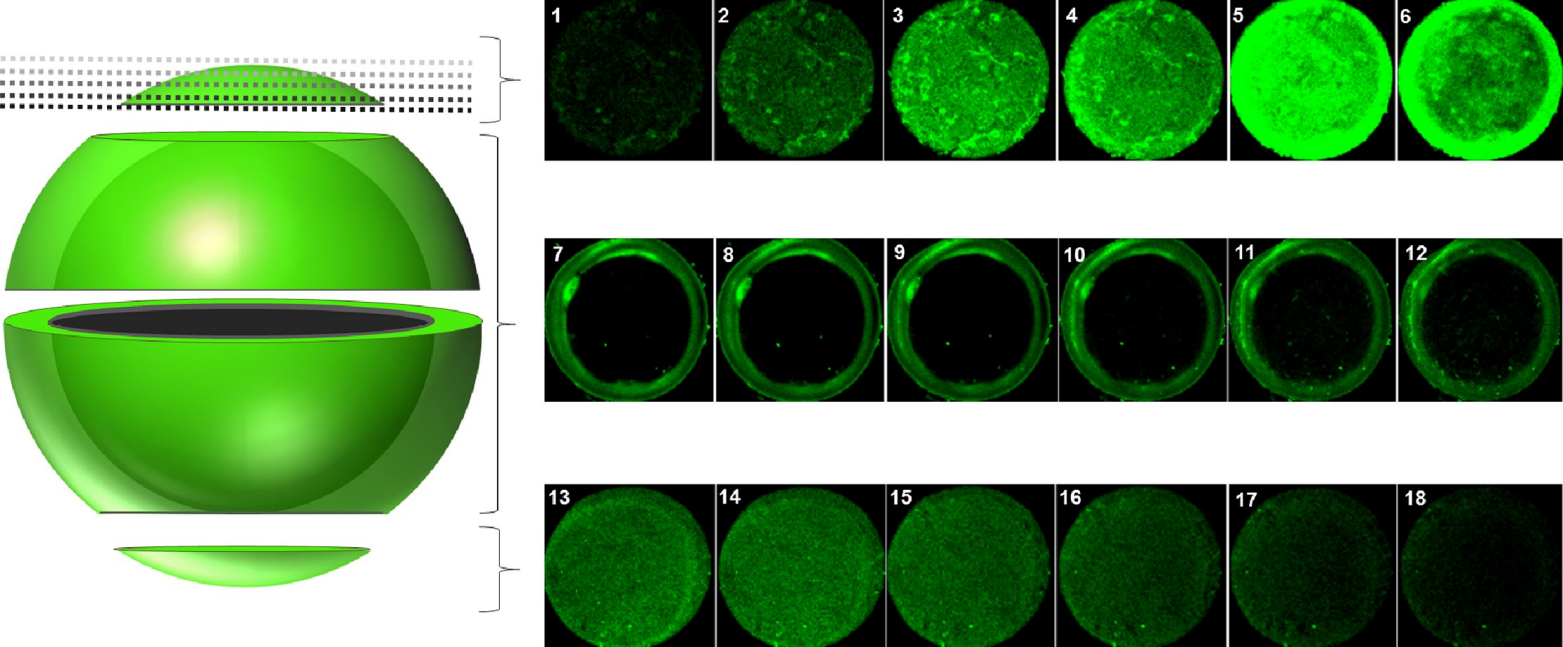
Confocal Z-stack image sequence of a denuded oocyte. Images of a denuded oocyte co-incubated with BHV1 for 24 h in a sequence of different focal planes (step size of 1μm) elucidating the presence of BHV1 (bright green dots) within the oocyte and on the outer face of the zona pellucida.

Denuded oocytes from negative control groups showed no presence of BHV1 after 1 h and 24 h incubation (Fig 2D). After BHV1 exposure, however, oocytes exhibited positive-BHV1 gC glycoprotein labeling, demonstrating the presence of this virus outside the ZP after 1 h incubation (Fig 2E), as well as inside the oocytes after 24 h incubation (Fig 2F). BHV1 was also evidenced inside denuded oocytes through the z-stack images, as well as on the ZP surface (Fig 4). A primary monoclonal antibody that binds to glycoprotein C of the BHV1 viral envelope enables the labeling of the viral structural proteins.

## Discussion

This study provides the first evidence of BHV1 within the COCs and denuded oocytes after 24 h incubation. The oocytes´ infection occurred in the presence of intact ZP, and thus BHV1 can cross the intact ZP of bovine oocytes after 24 h of co-incubation regardless of the presence of *cumulus* cells. BHV1 was also found in the cytoplasm of *cumulus* cells from all COCs exposed to this virus. After 1 h incubation, however, BHV1 presence was limited to the more peripheral region in the *cumulus* cells.

Herein, the detection of BHV1 was made using a primary monoclonal antibody that binds to glycoprotein C present in the BHV1 viral envelope. On this basis, BHV1 immunolabelling was identified in cells closest to the ZP, as well as inside the oocyte from infected COCs after 24 h exposure. It is important to emphasize that BHV1 completes its replicative cycle in approximately 18–20 h [2]. A 24 h incubation period was therefore enough for BHV1 to complete its replication and cross the intact ZP.

As supported by our findings, Silva-Frade et al. [25] reported that BoHV-5 was able to pass through the intact ZP of bovine oocytes. On the other hand, Vanroose et al. [11] concluded that intact ZP acts as an effective barrier to the entry of BHV1 into the bovine oocyte. However, these authors evaluated whether or not there was an infection of embryonic cells in BHV1-exposed embryos. It is noteworthy that bovine oocyte ZP presents approximately 1,511 pores, and they are large enough to allow the entry of BHV1 [23].

There must be interactions between the virus and the host cell to guarantee the success of virus replication within that cell. Accordingly, viral infection results in disordered cellular processes that can trigger apoptosis [30]. The latter, in turn, can be used by organisms as an antiviral defense. As a result, many viruses are able to modulate the apoptotic pathways of the host cell [31,32], preventing cell death and prolonging infections that, in the end, enables successful viral replication [33,34]. For instance, Silva-Frade et al. [35] concluded that BoHV-5 was able to suppress specific apoptotic pathways in infected bovine oocytes. However, BoHV-5 infection did not compromise the embryonic development as commonly described for BHV1 [11,18,22–24]. In bovine kidney (MDBK) cells, BHV1 was able to induce apoptosis slightly after penetration and then inhibited the apoptotic process [36]. The blocking of caspase activation by the virus itself increased BHV1 replication [37].

Indeed, many mechanisms involved in cell survival during viral infection remain unclear. Our findings, however, bring new understandings regarding the relationship between oocytes and BHV1, which may provide explanations for the impacts of BHV1 mentioned in several studies in the in vitro fertilization process and in embryonic development [11,18,22,23,38]. Further studies are necessary to clarify the reproductive failures due to viral infection, including temporary infertility and embryonic death.

BHV1 was also detected in the ZP layers, corroborating the findings previously described by Vanroose et al. [23,24]. Even though the International Embryo Transfer Society recommend a serial trypsin wash as an attempt to remove/inactivate the bovine herpesvirus 1 from embryos, their persistence in adherence with ZP even after the wash have been reported in several studies [9,39–42]. Similarly, Tsuboi et al. [9] detected BHV1 in cumulus cells even after trypsin washes by indirect immunofluorescence.

Furthermore, a partial disintegration of the cumulus cells was observed under light microscopy, especially in COCs after 24 h co-incubation with BHV1. This can be explained by the cellular lysis resulting from viral replication, known as the cytopathic effect. This effect is characterized by the ballooning of cells and the rise of intranuclear inclusions, which are characteristic of herpesvirus. The cytopathic effect was also mentioned by Tsuboi and Imada [10] in the oocytes of cumulus cells matured in vitro in the presence of BHV1, and by Vanroose et al. [11] due to the rounding of cumulus cells and concurrent failure to produce a confluent monolayer. In accordance with a recently published study that detected BHV1 in the cumulus cells of COCs from naturally infected cows [12], we have here evidenced the virus in cumulus cells after in vitro co-incubation for 1 h and 24 h.

## Conclusion

The detection of BHV1 inside oocytes with or without the *cumulus* cells has a great meaning for animal reproduction science. Our findings suggest new insights regarding the reproductive failures commonly shown in BHV1 infected cows.

## Supporting information

S1 Text Virus neutralization assay. (DOCX)

S2 Text Raw data of the experiment. (DOCX)
