# Correction: Bovine herpesvirus 1 can cross the intact zona pellucida of bovine oocytes after artificial infection

**DOI:** 10.1371/journal.pone.0222645

**Published:** 2019-09-12

**Authors:** Vanessa Lopes Dias Queiroz-Castro, Eduardo Paulino da Costa, Saullo Vinicius Pereira Alves, Mariana Machado-Neves, José Domingos Guimarães, Lidiany Lopes Gomes, Stella Vieira Domingos, Caroline Gomides Ribeiro, Rebeca Toledo Caldas, Abelardo Silva-Júnior

The following information is missing from the Funding Statement: VLD Queiroz-Castro received fellowship from Coordination for the Improvement of Higher Education Personnel (CAPES) (grant number PROEX0577/2018 to VLD Queiroz-Castro).
