# Supplementary material for: Bovine herpesvirus 1 can cross the intact zona pellucida of bovine oocytes after artificial infection
Source: PLoS One. 2019 Jul 18;14(7):e0218963. doi: 10.1371/journal.pone.0218963 (PMC6638837; doi:10.1371/journal.pone.0218963)
Supplement: S1 Text — (DOCX) [file pone.0218963.s001.docx]

**S1 Text. Virus neutralization assay**

For the virus neutralization assays, all the serum samples were previously inactivated for 56°C during 30 minutes. A 96-well microplate were utilized, in which, from the columns 5 to 11, the A-row were composed of 50µL of bovine kidney (MDBK) cells + 100µL of Minimum Essential Medium(MEM) and 50 µL of serum sample. The B-row well receive 50µL of MEM + 50µL of MDBK cells + 50µL of serum sample and 50µL of BHV1 at 100 TCID_50_ (Tissue Culture Infective Dose). The same procedure utilized for the B-row were performed for the C to H-row, by adding 50µL of the B well and following a serial dilution of the serum samples at the basis 2. The entire column 12 represents the negative control of the neutralizing assay, in which the well received 50µL of MEM + 50µL of MDBK cells + 50µL of fetal bovine serum and 50µL of BHV1 at 100 TCID_50._ The column 1 from A to G row, the well were composed of 50µL BHV1 at 100 TCID_50_ + 100µL of MEM and 50µL of MDBK cells. The wells from columns 2 to 4 were filled with the same amount of the reagents explained for column 1, except for the different virus concentration of 10^1^ TCID_50_, 10^0^ TCID_50_, 10^-1^ TCID_50_, respectively. Finally, the well from H1 to H4 were composed of 150µL of MEM + 50µL of MDBK cells.
