# Supplementary material for: Bovine herpesvirus 1 can cross the intact zona pellucida of bovine oocytes after artificial infection
Source: PLoS One. 2019 Jul 18;14(7):e0218963. doi: 10.1371/journal.pone.0218963 (PMC6638837; doi:10.1371/journal.pone.0218963)
Supplement: S2 Text — (DOCX) [file pone.0218963.s002.docx]

**S2 Text. Raw data of the experiment**

For the entire study, we obtained a total of 2,844 oocyte, which were aspirated from seropositive animals. From them, two groups were defined: 1,191 for the study of 1 h co-incubation and 1,653 for the study of 24 h co-incubation.

For the 1 h incubation study, 608 out of 1,191 oocytes were co-incubated with BHV1 in an incubator at 38.5 °C and 5% CO_2_, from them, 312 *cumulus*-oocyte complexes (COCs) and 296 denuded oocytes (without the presence of *cumulus* cells). Then, 583 oocytes, or 273 COCs and 310 denuded oocytes composed the negative control of the 1 h incubation study, in which they were subjected to the same protocol, except for exposure to BHV1.

Later on, all the oocytes were morphologically assessed by stereoscopic microscope. So, 281 out of 312 COCs (90%) co-incubated with BHV1 showed partial disintegration of *cumulus* cells (cytopathic effect), while none control COCs showed partial disintegration of *cumulus* cells. Furthermore, all (100%) denuded oocytes exposed to BHV1 and control denuded oocytes showed no morphological changes.

After the morphological assessment, all 1,191 oocytes were processed for immunofluorescence assays and evaluated using the confocal laser scanning microscopy. As expected, oocytes from negative control (n=583) did not present positive-BHV1 gC glycoprotein labeling. Conversely, all COCs co-incubated for 1 h were BHV1 positive. For COCs, the virus was detected in the cytoplasm of *cumulus* cells, especially in those located peripherally, while denuded oocytes presented immunelabelling restricted to the zona pellucida.

For the study of 24 h co-incubation, 830 oocytes (425 COCs and 405 denuded oocytes) out of 1,653 composed the group that was exposed to the BHV1 for 24 horas in an incubator at 38.5 °C and 5% CO_2_. Then, the control group of 823 oocytes were subjected to the same protocol, except for exposure to BHV1. We observed *cumulus* cells disintegration in all 24 h co-incubated COCs, while none COCs from the control group, in which the incubation were omitted, presented morphological changes, as well as those denuded oocytes.

Afterwards, all 1,653 oocytes were assessed by immunofluorescence assays and the oocytes from negative control (n=823) did not present positive-BHV1 gC glycoprotein labeling. However, the virus was detected in the cytoplasm of *cumulus* cells from all 425 co-incubated COCs. The BHV1 gC glycoprotein immunolabelling were observed in the cytoplasm of *cumulus* cells closest to the ZP and inside the oocyte in all COCs and denuded oocytes co-incubated with the virus for 24 h.
